# Data to improve air quality environmental justice outcomes in South Chicago

**DOI:** 10.3389/fpubh.2022.977948

**Published:** 2022-09-06

**Authors:** Tara Illgner, Nikita Lad

**Affiliations:** ^1^Department of Environmental Sciences, College and Graduate School of Arts and Sciences, University of Virginia, Charlottesville, VA, United States; ^2^Department of Environmental Science and Policy, College of Science, George Mason University, Fairfax, VA, United States

**Keywords:** air pollution, particulate matter, marginalized communities, industrial facilities, air-quality monitors, environmental justice, South Chicago, PM2.5

## Abstract

This brief provides data for policy changes on the intersection of polluting industrial facilities, marginalized communities, and the dearth of air-quality monitors in those communities of South Chicago. The allocation of air-quality monitors is determined by the Illinois Environmental Protection Agency (IEPA). Our data demonstrate that the air quality and subsequent health of Chicago's marginalized communities are chronically threatened by the expansion of polluting industries coupled with inadequate air-quality monitoring. The methods of data analysis presented here can be similarly applied to analyze air-quality disparities in communities beyond Chicago, including those that have been victims of environmental injustice stemming from redlining. Such data can be used to support Environmental Justice (EJ) advocacy and goals by informing remedial actions and policies of regulatory bodies, such as the IEPA, to reduce disproportionate air-quality burdens through increased monitoring. The generated maps may be used to direct plans for assessing and mitigating the effects of air pollution on human health.

## Key findings

- Polluting industrial facilities are disproportionately crowded in high Social Vulnerability Index (SVI)^1^ areas of Chicago with a significantly positive correlation of r^2^ = 0.94.^2^
- Over 60% of Chicago's industrial sites are located in areas with the highest SVI scores.
- Particulate matter (PM) pollution monitors for particles with diameters ≤ 2.5 μm (PM2.5), are lacking in most of the vulnerable and overburdened industrial hotspots.

## Background

The systemic division entrenched by the U.S. federal government's “redlining” program of 1933, ensured the marginalization of mostly Black communities (1). These communities are known as environmental justice (EJ) communities due to disproportionate environmental stress, including the overburden of toxic contaminants in their residential areas coupled with poor access to healthcare. South and Southeast Chicago EJ communities continue to face disproportionate and rising pollution exposure from the dense crowding of industrial facilities. In 2021 resident EJ activists protested deteriorating air quality, resulting in a hunger strike and four arrests (2, 3). Chicago's EJ communities are encircled by polluting industrial plants, increasing residents' exposure to particulate matter (PM) air pollution, which is linked to serious health consequences. Furthermore, adequate air-quality monitors are lacking in the communities most burdened with polluting facilities. This article focuses on PM air pollution.

Inhalable PM pollution, particularly of particles with diameters ≤ 2.5 μm (PM2.5), is known to contribute to poor health outcomes including asthma, cancer, obesity, diabetes, low birth weight, stroke, depression, and cardiovascular diseases (4, 5). Communities near PM-polluting facilities also report headaches, nausea, and trouble breathing (6). Since the start of the COVID-19 pandemic, comorbidities attributed to PM exposure combined with poor access to healthcare for Black residents have contributed to a COVID-19 death rate that is *twice* the national average (7).

## Methods

**Figure 1:**
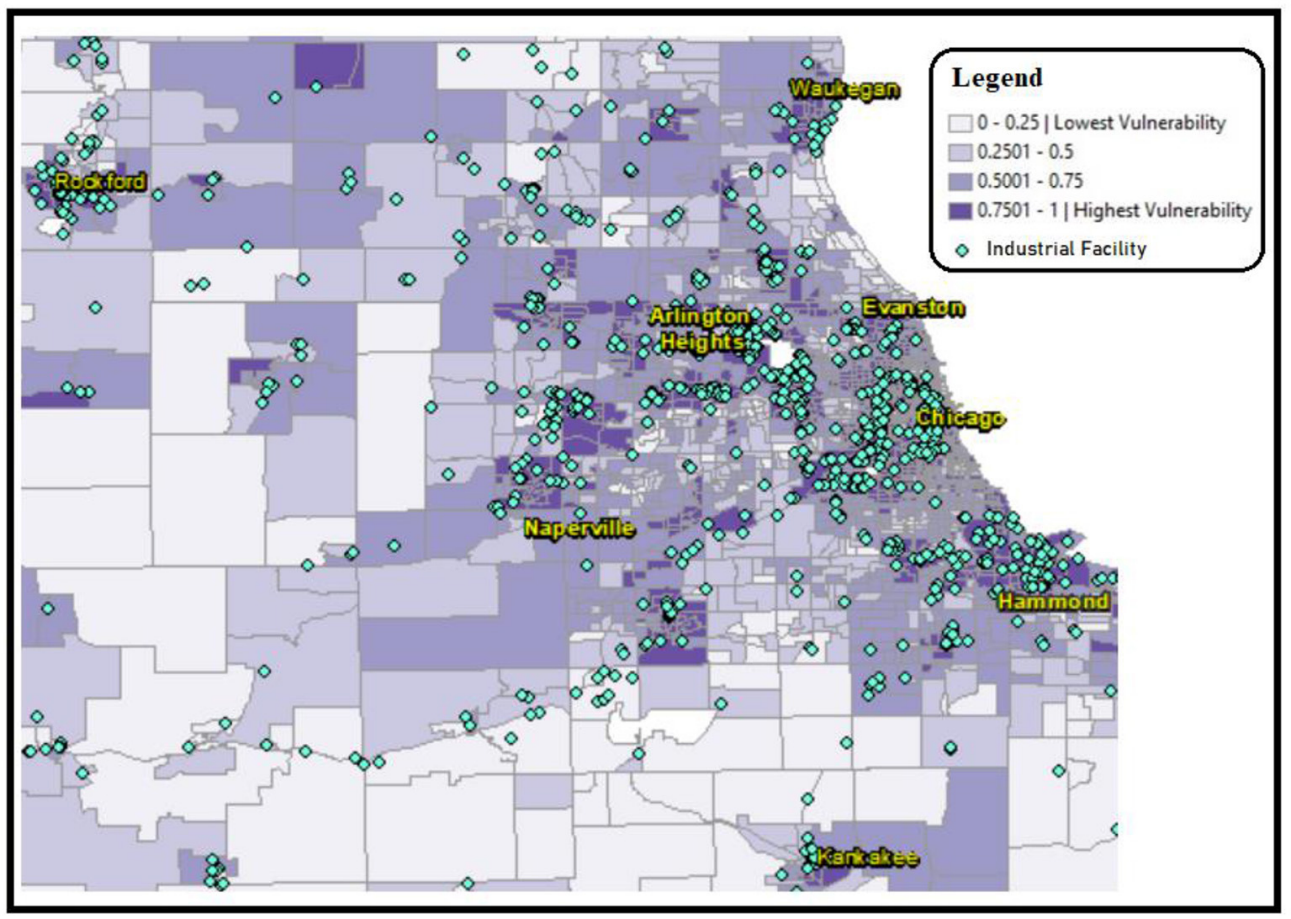
CDC census tract Social Vulnerability Index (SVI) represented in shades of purple, overlaid with industrial facility sites represented with turquois diamonds. This map shows a prevalence of industrial facilities located in areas of high SVI scores. Industrial site source data retrieved from Commission for Environmental Cooperation (CEC) (10), SVI data retrieved from CDC data (2018), analysis performed using ESRI ArcMap 10.8.

Using geographic information system (GIS) analysis in ArcMap 10.8, we mapped the US Centers for Disease Control Social Vulnerability Index (SVI) census tract scores for the city of Chicago and surrounding areas within 100 miles of Chicago (8). The SVI considers fifteen factors that contribute to harming the health and wellbeing of residents in a given census tract, including poverty, lack of access to transportation, linguistic isolation, and crowded housing (9). The census tract map (Figure 1) displaying SVI scores, represented in shades of purple, was then overlaid with the locations of polluting industrial facilities, represented in turquois (10).

**Figure 2:**
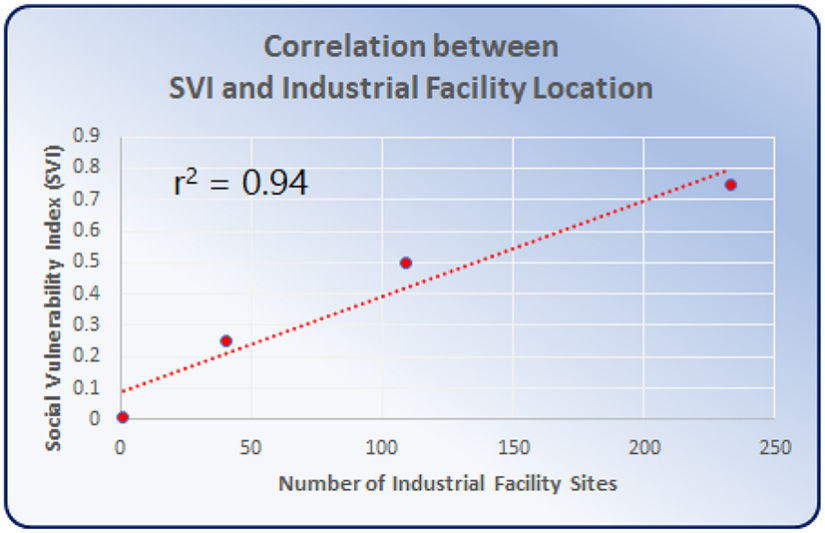
Data from Figure 1 used to generate correlation analysis for SVI scores and number of industries within census tracts. The trend reveals a strong correlation (r^2^ = 0.94), indicating a very high likelihood of industrial facility location in the most socially vulnerable census tracts, compared with surrounding areas.

In addition to the visual confluence of polluting industrial facilities and high SVI scoring areas provided in Figure 1, we performed a correlation regression analysis of the data (Figure 2), using Microsoft Excel. The data analyzed 383 industrial facilities emitting PM2.5 within 1,315 census tracts of Cook County, and SVI scores between 0 and 1 (10). The CDC labels SVI scores between 0.75 and 1 as “highest vulnerability” and scores between 0 and 0.25 as “lowest vulnerability.” The CDC did not label the intermediate scores. Therefore, we labeled scores between 0.5 and 0.75 as “moderate” and those between 0.25 and 0.5 as “low.” Figure 2 displays the correlation analysis results of the 4 census tract SVI status labels and the corresponding number of polluting facilities located under each label. Table 1 displays the data used for Figure 2, which shows significantly more industrial sites in areas of “highest vulnerability” (SVI score of 0.75–1).

**Table 1:** Displays the data used for the correlation analysis in Figure 2: “Status” and “Number of Industrial Facilities.”

**Census tracts**				**Per status**	
**Total**	**SVI score**	**Status**	**Number of census tracts**	**Number of Industrial facilities**	**Number of active PM2.5 monitors**
1,315 Census tracts within Cook County, Chicago	0.75–1	High	565	233	4
	0.5–0.75	Moderate	501	109	3
	0.25–0.5	Low	210	40	0
	0–0.25	Lowest	39	1	0

The resulting linear regression in Figure 2 produced a correlation coefficient (r) of 0.97, an r2 value of 0.94, and a confidence interval of 0.8–1.14. The table also displays the “Number of PM2.5 Monitors” throughout the census tracts for each “Status” label.

**Figure 3:**
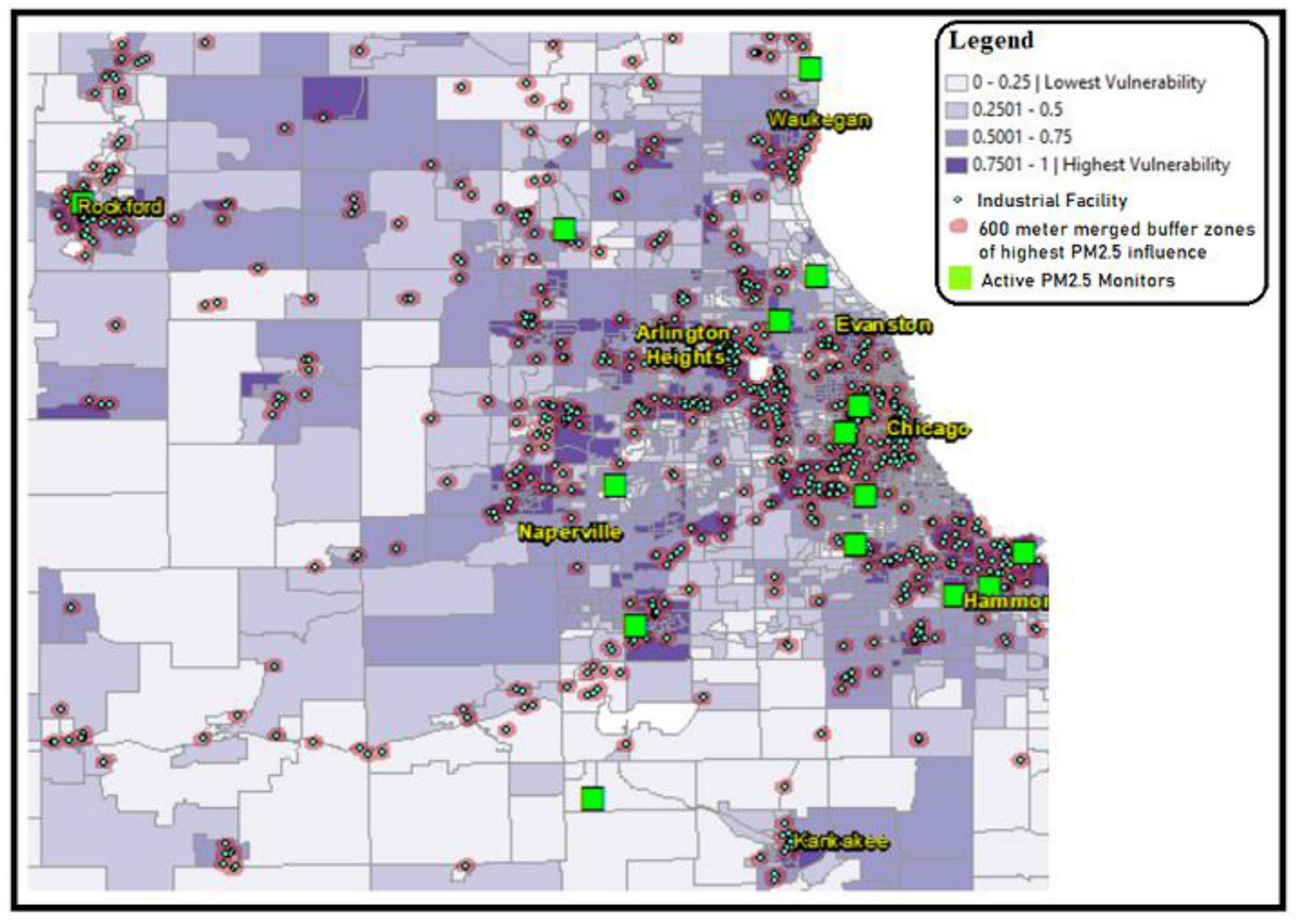
CDC census tract SVI scores represented in shades of purple, overlaid with industrial facility locations represented with turquois diamonds. This map shows a prevalence of industrial facility sites located in areas of high SVI scores. The green squares indicate the locations of the EPA active PM2.5 monitors as of 2020. The red areas indicate the EPA radius of PM2.5 pollution influence (12). Authors recommend additional PM2.5 monitors for areas that represent with a confluence of high SVI scores (purple), PM2.5 radius of influence, and lack of centrally located insufficient PM2.5 monitoring. Industrial site source data retrieved from Commission for Environmental Cooperation (CEC) (10), SVI data retrieved from CDC data (2018), PM2.5 monitor sites retrieved from EPA, analysis performed using ESRI ArcMap 10.8.

In October 2021, the Illinois EPA (IEPA) requested public comments on their new proposal for additional PM2.5 monitoring locations. In response, our team created a new map using ArcMap adding the locations of the existing and latest active PM2.5 monitor locations (green squares) according to the EPA's GIS monitor locations in Chicago (11), contrasted with persistently neglected areas of high industrial crowding (red). The red areas were created following the EPA report (12) designating a PM2.5 radius of influence surrounding 600 m from the source of pollution, such as industrial facilities (turquois diamonds in Figure 1). We used ArcMap to assign a 600-meter buffer zone around the industrial facilities, and then merged overlapping buffer zones to create the red areas in Figure 3. Beyond 600 m, PM2.5 concentration declines through dilution with ambient air (13). Accordingly, the areas in Figure 3 with overlapping factors of (a) high SVI (purple), (b) PM2.5 radius of influence (red), and (c) lack of centrally located active PM2.5 monitor represent our recommended areas for increased monitoring. To our knowledge, this is one of the first studies that considers all the three key features for recommendation of air quality monitoring sites.

## Results

Figure 1 displays the disproportionate crowding of industrial facilities (turquois) in the areas with the highest SVI scores (purple). Analysis of mapping data revealed that the highest SVI scoring census tracts are home to over 60% of Chicago's industrial sites. The data used for Figure 2 showed significantly more industrial sites in areas of “highest vulnerability” (SVI score of 0.75–1). The resulting graph in Figure 2 shows a very high correlation (r^2^ = 0.94) between SVI score and industrial sites. Correlation r^2^ values fall between 0 and 1, where values closer to 1 (such as 0.94) are indicative of a stronger correlation that is extremely unlikely to be accidental. Therefore, the correlation displayed in Figure 2 demonstrates that the number of industrial sites is highest where SVI scores are also highest. Conversely, a correlation analysis between SVI score and number of PM monitors resulted in an r^2^ value of 0.88, which is lower than the correlation between SVI score and industrial sites. Figure 3 displays active EPA PM2.5 monitoring (green squares) in relation to SVI scores (shaded purple census tracts), and the locations of industrial facilities (turquois diamonds). In Figure 3, our policy recommendation is to adjust the upcoming IEPA State Implementation Plan (SIP) to include increased monitors in neglected hotspots that exhibit the convergence of (a) high SVI scores (purple), (b) PM2.5 radius of influence (red), and (c) lacking centrally placed PM monitors. These hotspots include areas in or near Waukegan, Arlington Heights, Chicago, Naperville, Hammond, Rockford, and Kankakee.

## Discussion

The results of these analyses (Figures 1–3) demonstrate the disproportionate crowding of polluting facilities in high SVI areas of Chicago. This crowding overburdens low-income areas and areas with people of color, forcing already under-resourced communities to combat toxic contaminants in their water and air. In 1997, the U.S. Environmental Protection Agency (EPA) set National Ambient Air Quality Standards (NAAQS), which set limits for concentrations of six principal air pollutants that are deemed harmful to public health and the environment, including PM2.5 (14). While overall PM levels have declined nationally since the implementation of NAAQS, local hotspots persist in EJ communities, such as those identified here in South and Southeast Chicago. Chicago's long and fraught history of tense and unbalanced relationships between high PM-emitting industries and EJ communities residing near these industries has resulted in persistent health stresses upon the most vulnerable residents. While our calculated correlation between SVI scores and PM monitoring is also significant (r^2^ = 0.8), it is less than the correlation between high SVI scores and industrial sites (r^2^ = 0.94). In other words, the likelihood of industrial exposure in high SVI scoring areas is higher than the likelihood of sufficient monitoring. Therefore, our policy recommendation for the Illinois SIP is to allocate a commensurate increase in PM monitoring in high-scoring SVI areas.

Figure 3 represents our response to the EPA's existing locations for PM2.5 monitoring sites, where the green squares represent the sites of all active PM2.5 monitors. The notable lack of sufficient air-quality monitoring in high SVI areas with dense industrial crowding, as shown in Figure 3, results in an inaccurate representation of the air quality that many of Chicago's most vulnerable residents face, which further contributes to ill-informed decision-making and policies for the permitting and placement of future polluting sources, such as industrial facilities. According to the EPA (12), the PM air pollution is highest within 600 m of the source of emissions (i.e., industrial site) while concentrations decline with distance from the source. Areas in Figure 3 where overlap exists between high SVI score, PM2.5 radius of influence, and lack of centrally located, represent neglected PM2.5 hotspots. For these hotspots, our policy recommendation is for the Illinois SIP to assign more monitors to accurately assess the air-pollution burdens faced by the residents of these areas. At the policy level, increased monitoring may reveal areas of excessive PM concentrations above the U.S. EPA National Ambient Air Quality Standards (NAAQS) limits, which would trigger the need for the IEPA to develop a new SIP to reduce localized excess PM concentrations, such as by halting permits to new nearby industrial developments.

## Conclusion

Overall, Figures 1–3 provide valuable data that can be used to support South Chicago residents' clean-air advocacy efforts for air-quality equality. This data can better inform state and local air-quality regulatory bodies, including the IEPA, about industrial crowding that leads to disproportionate harmful air-quality burdens in EJ communities. Policy changes by the IEPA can alleviate PM2.5 air pollution hot-spots. Disseminating this data increases awareness of the causes and consequences of combining industrial crowding with insufficient air-quality monitoring. Additionally, these maps can better inform policy-makers and healthcare providers to tailor care, support, and advocacy for these overburdened communities, as well as galvanize non-governmental organizations and communities in other US cities to similarly analyze the industrial burdens upon air quality in their area. Therefore, by increasing monitoring, decision-makers can improve policies and limitations of industrial plant placement to safeguard the health, welfare, and quality of life for EJ communities and their environment in Chicago and beyond.

## Data availability statement

The original contributions presented in the study are included in the article/supplementary material, further inquiries can be directed to the corresponding author.

## Author contributions

TL and NL contributed to the conception of the topic. TL gathered the database and performed the GIS analysis. NL contributed to the writing of the manuscript sections. All authors revised, read, and approved the submitted version.

## Conflict of interest

The authors declare that the research was conducted in the absence of any commercial or financial relationships that could be construed as a potential conflict of interest.

## Publisher's note

All claims expressed in this article are solely those of the authors and do not necessarily represent those of their affiliated organizations, or those of the publisher, the editors and the reviewers. Any product that may be evaluated in this article, or claim that may be made by its manufacturer, is not guaranteed or endorsed by the publisher.

## Footnotes

^1^The U.S. Centers for Disease Control and Prevention (CDC) measures social vulnerability of an area's residents using the Social Vulnerability Index (SVI) score, which was used for this correlation analysis.

^2^Correlation r^2^ values range between 0-1. The closer the r^2^ value is to 1, the more strongly correlated are the observations.
